# Turn Waste Golden Tide into Treasure: Bio-Adsorbent Synthesis for CO_2_ Capture with K_2_FeO_4_ as Catalytic Oxidative Activator

**DOI:** 10.3390/molecules29061345

**Published:** 2024-03-18

**Authors:** Huijuan Ying, Chenglin Jia, Ganning Zeng, Ning Ai

**Affiliations:** 1 College of Chemical Engineering, Zhejiang University of Technology, Hangzhou 310014, China; yinghuijuan@zjut.edu.cn (H.Y.); 202105490407@zjut.edu.cn (C.J.); 2 College of Environment, Zhejiang University of Technology, Hangzhou 310014, China; gnzeng@zjut.edu.cn; 3 College of Biological, Chemical Science and Engineering, Jiaxing University, Jiaxing 314001, China

**Keywords:** bio-adsorbent, *Sargassum horneri*, oxidative pyrolysis, K_2_FeO_4_, CO_2_ capture

## Abstract

Converting *Sargassum horneri* (SH)—a harmful marine stranding that can cause golden tide—to highly porous bio-adsorbent material (via one-step catalytic oxidative pyrolysis with K_2_FeO_4_) can be a strategically useful method for obtaining low-cost materials suitable for CO_2_ capture. In this manuscript, the behavior of different mass ratios of K_2_FeO_4_/SH precursor acting on the surface physicochemical properties of carbon materials are reported. The results suggest that specific surface area and total pore volume first increased to the mass ratio of K_2_FeO_4_/carbon precursor, then decreased. Among the samples prepared, the highest specific surface area was obtained with a K_2_FeO_4_/SH precursor ratio of 1:4 (25%-ASHC), and the CO_2_ adsorption performance was significantly increased and faster compared with the original biochar. The fitted values of the three kinetic models showed that the double exponential model provided the best description of carbon adsorption, indicating both physical and chemical adsorption; 25%-ASHC also exhibited excellent cyclic stability. The improved CO_2_ adsorption performance observed after K_2_FeO_4_ activation is mainly due to the increase in material porosity, specific surface area, and the enrichment of nitrogen and oxygen functional groups.

## 1. Introduction

In recent years, the golden tide dominated by *Sargassum* has become a kind of emerging marine ecological hazard occurring frequently around the world. The ecological phenomenon whereby the floating brown genus *Sargassum* changes the color of seawater as a result of rapid growth or high concentration enrichment is known as a “golden tide” [1]. Since 2002, large-scale golden tide invasions have occurred along the Brazilian coast of the South Atlantic Ocean, the Caribbean coast, the west coast of Africa, the waters of the tropical island nations of the South Pacific Ocean, and the North Pacific coast, with devastating impacts on the local tourism economy, fishery resources, and the marine environment [2,3,4,5,6]. The state of Texas in the United States spends at least $2.9 million each year cleaning up *Sargassum* on beaches. In China, since 2012, large-scale floating and gathering of *Sargassum* has also occurred off the mouth of the Yangtze River, Shandong Province (to the east of Rizhao), and the coasts of Dalian and Jiangsu Provinces, gradually developing into a golden tide that has struck the country’s coastal cities [7,8,9,10,11]. Especially in Jiangsu’s seaweed culture area, due to the emergence of golden tide, the breeding rafts seriously collapsed and resulted in Nantong and Yancheng experiencing direct economic losses of up to more than $70 million. In 2017, a wide-scale golden tide of *Sargassum* occurred east of the Yellow Sea, triggering the rare phenomenon of “three tides”—golden tide, green tide, and red tide, and the distribution area of golden tide even exceeded the area of green tide during the same period. This indicates that the golden tide has become the second large-scale seaweed disaster in China’s offshore after the green tide. This kind of disaster will cover a large area of the sea and seriously affect the growth of other algaes. On the other hand, the aggregation and decomposition of *Sargassum* will generate toxic and hazardous substances, which will easily cause pollution of the water environment, aggravate eutrophication and hypoxia in the water body, and have the potential of triggering the red tide of certain microalgae. In serious cases, it may also affect the marine planktonic ecosystem, making demersal neritic fish and Crustacea the most affected groups [9,12]. In addition, the massive accumulation and stranding of algae in the shallows and along the shoreline during the decline period not only hinders vessel navigation and endangers the shallow water aquaculture industry and coastal ecosystems, but also jeopardizes the safety of marine ecosystems and triggers the growth of *Vibrio* vulnificus and other related problems, which in turn threaten human health. Some modelling studies have speculated that in the future, the phenomenon of golden tides similar to the one in 2017 in China’s offshore is also likely to recur, or even become more serious, along with global warming and the intensification of nutrient inputs with riched nitrogen and phosphorus from offshore aquaculture [9,13]. Therefore, it is necessary to explore research on strategies to increase the added value of *Sargassum* in order to reduce its detrimental impacts.

The composition of *Sargassum* mainly consists of polysaccharides such as lignin, cellulose, hemicellulose, and alginate. In recent years, the use of its biological properties to prepare various biomass-based materials has been reported, with applications in various fields such as medicine [14], energy storage [15], and adsorption [16,17]. Especially in the field of adsorption, *Sargassum* has been favored by scholars for its complex cell wall structure, abundant phycobiliprotein, fucoidan, sulphated seaweed polysaccharides and high concentration of carboxyl groups, and other biological properties that are conducive to rapid adsorption [18]. Gonzalez Fernandez et al. [19] suggested that the shape of *Sargassum*, the ideal surface charge density, and the presence of abundant hydroxyl as well as –COOH groups are essential for the effective enhancement of heavy metal adsorption performance. Jafarian et al. [20] developed a sustainable and cost-effective *Sargassum*-based cationic dye adsorbent, indicating that *Sargassum* has a fibrous structure, and its matrix contains a variety of polysaccharides and a high concentration of active elements (Na, Mg, N, S, P, etc.), which are suitable for adsorption. Along with the increase in greenhouse effect, there are also studies on the application of *Sargassum* to CO_2_ adsorption, mainly focusing on the alkali metal activation process, especially KOH, which has gained attention due to its ability to obtain a large specific surface area. Ding et al. [21] prepared algal-based activated carbon by KOH modification, taking advantage of the carbon-, oxygen- and sulphur-enriched *Sargassum*, and the results showed that the high specific surface area and the oxygen- and sulphur-containing functional groups were the main factors to improve its adsorption performance. Our previous work also found that using nitrogen- and potassium-enriched *Sargassum* as the precursor to prepare carbonaceous adsorbent can reduce the dosage of KOH with good CO_2_ adsorption performance [22]. However, it is well known that the disadvantages of using KOH as an activator are also quite obvious, such as higher temperature, higher energy consumption, more serious destruction of the biomass self-template, and the corrosive problem of the equipment is difficult to solve with larger dosage. Therefore, looking for a greener activator is an urgent problem to be solved at present.

As an efficient green biocide, potassium ferrate (K_2_FeO_4_) is commonly used in the purification of drinking water [23]. It is significantly more environmentally friendly than alkali metal activators such as KOH, and the lower K content of K_2_FeO_4_ protects the biomass from the self-template effect and prevents over-etching [24]. Consequently, K_2_FeO_4_ has shown distinct advantages in the development of other biomass-based carbon-based materials since 2017. It is widely used for the removal of metal ions, organics, and dyes from wastewater [25,26,27] as it not only has the pore-forming ability of the potassium-based component, but also has the catalytic function of graphitization of the iron-based component, which can synergistically regulate the pore structure and enhance the degree of graphitization of the carbon material [28]. However, most of the existing studies ignored that K_2_FeO_4_ has a stronger oxidizing ability than potassium permanganate (KMnO_4_), which can be used for oxidative modification of biomass-based carbon materials to increase the number and type of surface functional groups in order to improve the adsorption capacity. Meanwhile, the presence of Fe can not only play a catalytic role in lowering the reaction temperature and reducing the reaction time, but also improve the stability of the carbon material through the metal modification of iron. Inspired by our previous work, the addition of Fe did significantly accelerate the activation of K and reduced the KOH loading and reaction temperature. The carbon materials synthesized from Fe(NO_3_)_3_/KOH as co-activator had higher specific surface area and better CO_2_ uptake capacity than those activated only by KOH [29,30]. Therefore, K_2_FeO_4_ will be an effective activator for CO_2_ bio-adsorbent preparation. Nevertheless, there are few studies on the synthesis of biomass-based carbon materials using K_2_FeO_4_ for CO_2_ capture.

In this study, the catalytic, activating, and oxidizing properties of K_2_FeO_4_ were fully utilized. Combining the sustainable and low-cost advantages of abundant *Sargassum horneri* (SH, a kind of *Sargassum*) reserves, fast growth rate, easy cultivation, and harvesting, the one-step pyrolysis and oxidation activation with K_2_FeO_4_ was used to produce self-doped SH-based carbon materials (ASHC). Compared with the traditional two-step process of carbonization followed by activation, one-step pyrolysis has the advantages of less time and energy consumption, which reduce the production cost. This paper focus on the different mass ratios of K_2_FeO_4_/SH precursor to the physical and chemical properties of carbonaceous material, and three adsorption kinetic models were fitted and analyzed under different adsorption temperatures. Cyclic adsorption–desorption behavior was evaluated to assess the feasibility of the carbonaceous materials for long-term applications. Turning seaweed into treasure and applying it in carbon dioxide capture provides a pathway for simultaneously solving two global problems—the golden tide and the greenhouse effect. This study can also provide an important reference for achieving green, large-scale, and low-cost production of biomass porous carbon solid adsorbent materials.

## 2. Results and Discussion

### 2.1. Characterization of Adsorbents

#### 2.1.1. BET Analysis

**Figure 1:**
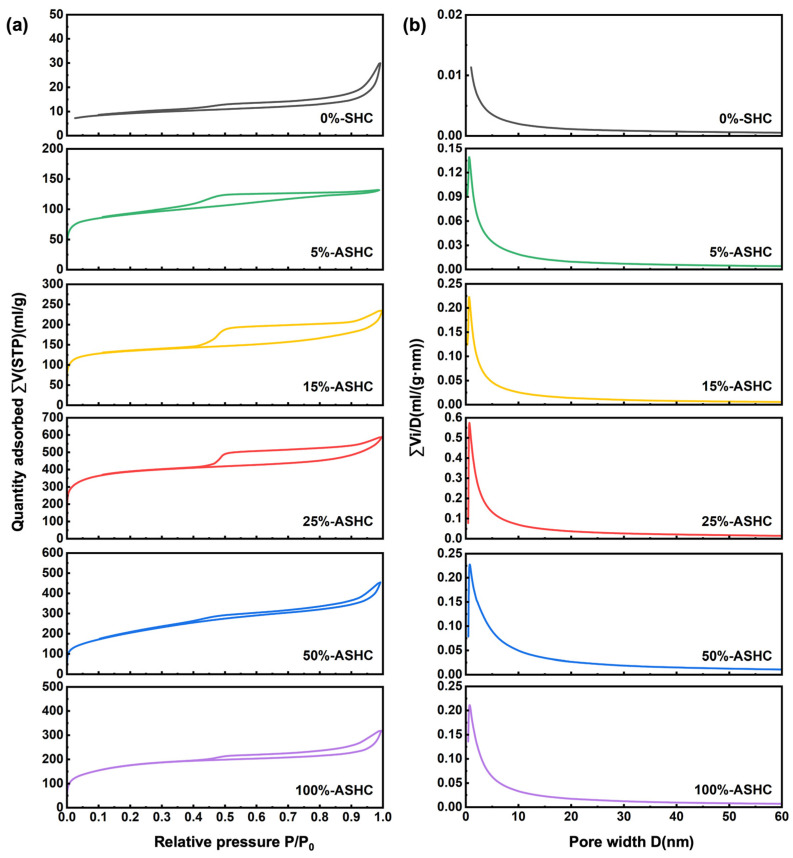
(**a**) N_2_ adsorption–desorption isotherms of samples; (**b**) corresponding pore size distribution of samples.

To illustrate the contribution of K_2_FeO_4_ to formation of pore structure, BET analysis was performed on samples with loading increase. As shown in Figure 1, the adsorption–desorption isotherms of the five activated carbon materials exhibited typical type I isotherms in accordance with the IUPAC standard. There was a clear adsorption inflection point at low relative pressure (P/P_0_ < 0.4), suggesting that the adsorbent has a rich microporous structure [31]. All isotherms exhibited H4-type hysteresis cycles in the region of high relative pressure (P/P_0_ > 0.4), the presence of which signaled that the micropores were also accompanied by mesopores and macropores. In addition, the pore size distribution curves indicate that the obtained ASHCs have a large number of ultra-micropores (<0.7 nm), which will play the critical role in CO_2_ adsorption at low pressures [32]. All ASHCs have a narrow microporous pore size distribution centered on about 0.7 nm. However, different amounts of activators cause different activation effects on all the samples, resulting in a big difference in micropore volume and also a big difference in total pore volume and specific surface area, which may lead to different CO_2_ adsorption of different samples. As shown in Table 1, we can see that all the activator-added adsorbents showed a significant increase compared to the pristine biochar adsorbent. Specific surface area, total pore volume, and microporous volume increased and then decreased with the addition of an activator, showing a maximum at 25wt% addition; 5%-ASHC had the smallest average diameter of micropores, but its lower total micropore volume possibly make it poor at carbon dioxide adsorption. On the other hand, 50%- and 100%-ASHC may have partially collapsed char wall structure because of the over-activation by K_2_FeO_4_ [33,34]. Overall, 25%-ASHC exhibited the largest specific surface area (1245 m^2^·g^−1^), pore volume (0.8827 cm^3^·g^−1^), and micropore volume (0.5682 cm^3^·g^−1^), as well as the highest percentage of micropore volume (0.6437 cm^3^·g^−1^)—almost 40 times, 20 times, and 25 times that of 0%-SHC. This is because appropriate K_2_FeO_4_ activation can significantly increase the BET surface area and total pore volume of SH, which will contribute to promoting CO_2_ adsorption. As Gong et al. first summarized in 2017 [28], K_2_FeO_4_ was used as both the activating agent (K) and catalyst (Fe species) to regulate pore structure and catalytic graphitization of SH, according to Equation (1).

(1) $${4K}_{2}{FeO}_{4}+{10H}_{2}O\to 8KOH+{4Fe(OH)}_{2}+{3O}_{2}$$

The mechanism mainly demonstrated the pore formation as KOH activation with Equations (2)–(5) in Wang and Kaskel’s report [35]. KOH starts by reacting at 400–600 °C to form the intermediate products K_2_O and K_2_CO_3_ (Equations (2) and (3)). When the temperature is higher than 700 °C, K_2_CO_3_ partially decomposes into K_2_O and CO_2_ (Equation (4)). Meanwhile, the produced CO_2_, K_2_O, and K_2_CO_3_ further reacted with carbon to form CO and potassium metal (Equations (5)–(7)). The free potassium metal penetrates into the carbon lattice, causing the lattice to swell, which is then rapidly detached from the carbon organism, forming a pore structure [36].

(2) $$2KOH↔K_{2}O+H_{2}O$$

(3) $$6KOH+2C\to 2K+{3H}_{2}+{2K}_{2}{CO}_{3}$$

(4) $$K_{2}{CO}_{3}↔K_{2}O+{CO}_{2}$$

(5) $${CO}_{2}+C\to 2CO$$

(6) $$K_{2}{CO}_{3}+2C\to 2K+CO$$

(7) $$K_{2}O+C\to 2K+CO$$

The catalytic effect of K_2_FeO_4_ is mainly reflected in Equations (8)–(11). Fe(OH)_3_ is firstly converted to Fe_2_O_3_ at 400 °C (Equation (8)), which are all α-Fe_2_O_3_ at first; later, α-Fe_2_O_3_ is partially converted to γ-Fe_2_O_3_ above 500 °C (Equation (9)) and is further converted to Fe_3_O_4_ (Equation (10)). The reducing components further interact with Fe_3_O_4_ to form Fe (Equation (11)) [37]. The presence of iron oxide crystals was also detected in the subsequent XRD. As an efficient catalyst, Fe played an especially important role in decreasing the reaction temperature, accelerating the activation process, and catalyzing graphitization of SH. Moreover, it will not produce any contaminants during the preparation compared with other metals [38].

(8) $${Fe\left(OH\right)}_{3}\to FeO\left(OH\right)\to {Fe}_{2}O_{3}$$

(9) $${\alpha -Fe}_{2}O_{3}\to {\gamma -Fe}_{2}O_{3}$$

(10) $$3{Fe}_{2}O_{3}+(H_{2},CO,C)\to 2{Fe}_{3}O_{4}+{(H_{2}O,CO,CO}_{2})$$

(11) $${Fe}_{3}O_{4}+4\left(H_{2},CO,C\right)\to 3Fe+{4(H_{2}O,CO,CO}_{2})$$

#### 2.1.2. SEM and TEM Analysis

**Figure 2:**
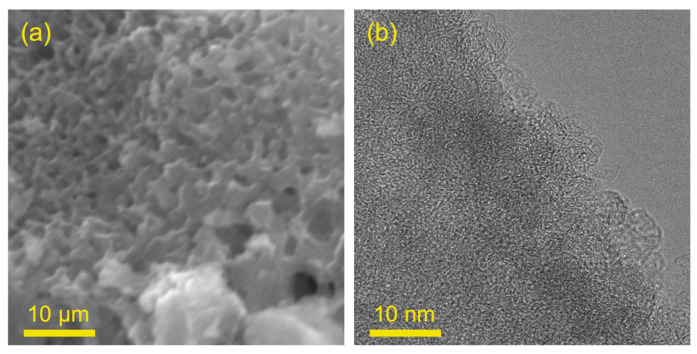
(**a**) SEM image of 25%−ASHC; (**b**) TEM image of 25%−ASHC.

The SEM image of 25%-ASHC was shown in Figure 2. We reported [30] the structural morphology of pristine SH, which mainly consists of axial tubular fibers with a dense structure; 25%-ASHC formed a dense honeycomb structure on the surface; this is because the dense structure of pristine SH was destroyed after activation; the etching of component K resulted in a large number of pores formed. By contrast, as shown in Figure S1a,b, 15%-ASHC had holes with smaller diameters on the surface, but they were fewer in number and more dispersed, leaving the holes disconnected from each other, while the surface roughness of the 50%-ASHC sample is reduced, which may be the result of excessive activation. These obtained results are consistent with BET analysis consequence. High-resolution transmission electron microscopy (TEM) was used to observe in more detail about 25%-ASHC as shown in Figure 2b. It can be observed that the 25%-ASHC has obvious characteristics of carbon material with flaky edges, along with a microporous structure, which is crucial for the CO_2_ adsorption performance of activated carbon as the kinetic diameter of CO_2_ is 0.33 nm; studies have shown that the adsorption rate of CO_2_ mainly depends on the volume of narrow micropores with pore size less than 0.8 nm [39,40]. Here, the micropore diameter of 25%-ASHC is 0.72 nm from BET analysis, consistent with TEM results.

#### 2.1.3. FTIR Analysis

**Figure 3:**
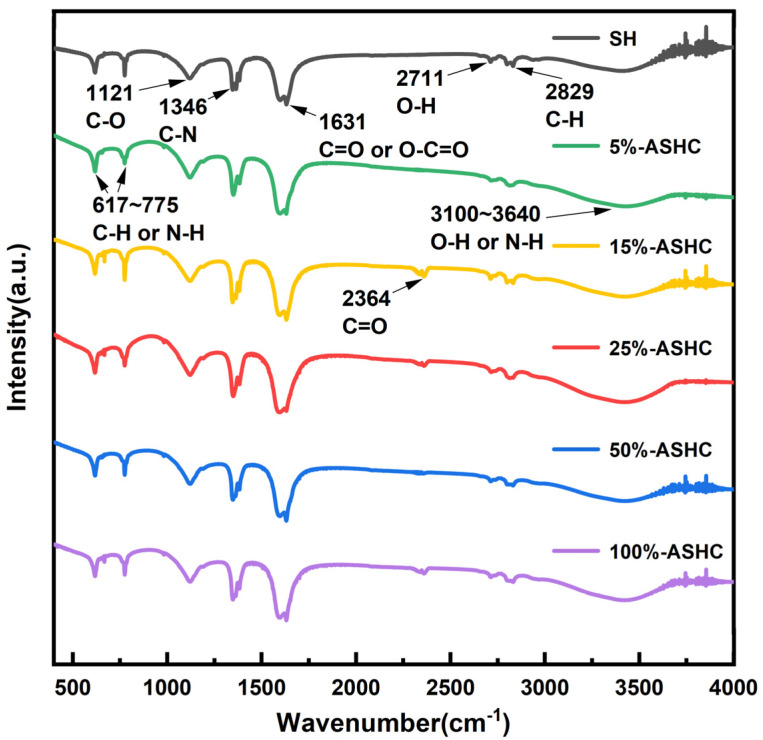
FTIR spectra of SHC and ASHCs.

The FTIR spectra of SHC and ASHCs are shown in Figure 3. A very broad peak appears at 3100~3640 cm^−1^ in all samples, which may be attributed to the O-H and N-H stretching vibration of carboxyl and amine groups, respectively. The strongest peak at 1631 cm^−1^ corresponds to carbonyl C=O stretching vibration of surface functional groups [27]. The peaks at 1121 cm^−1^ correspond to the stretching vibration of the C-O bond. These oxygen-containing functional groups are likely to be ketol or lactone groups due to the oxidation of K_2_FeO_4_, which are usually alkaline and beneficial for carbon dioxide adsorption. This was verified by subsequent XPS detection. Additionally, the weak adsorption peak at 617 cm^−1^ and 2829 cm^−1^ corresponds to a saturated C-H stretching vibration adsorption peak, while 775 cm^−1^ and 3450 cm^−1^ corresponds to the out-of-plane bending vibration of the N-H bond. The peaks at 1121 cm^−1^ correspond to the stretching vibration of the C-N bond. The presence of nitrogen-containing functional groups also supports the idea that SH are rich in protein, which will play a self-doping role during pyrolysis to enhance the CO_2_ adsorption [41]. Additionally, the peak observed at 2357 cm^−1^ may be due to the adsorption of CO_2_ by the sample during storage. As can be seen in Figure 3, there is no CO_2_ adsorption peak on the surface of the original carbon and 5%-ASHC, but there is the rest of the activated carbon, which indicates that the activation of an appropriate amount of potassium ferrate can indeed improve its adsorption capacity for carbon dioxide.

#### 2.1.4. XPS Analysis

**Figure 4:**
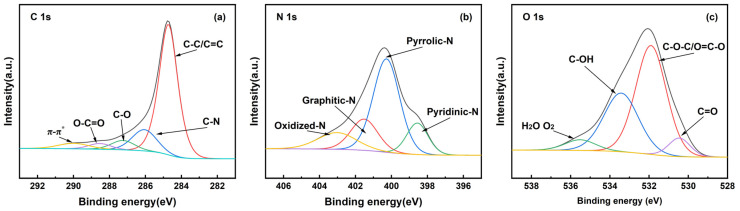
XPS patterns of 25%-ASHC (**a**).C1s spectrum; (**b**) N1s spectrum; (**c**) O1s spectrum.

Three peaks of 25%-ASHC at 285, 400, and 532 eV were observed and attributed to carbon (C 1 s), nitrogen (N 1 s), and oxygen (O 1 s), respectively, as shown in Figure S2. Figure 4 shows fitting of C 1 s, O 1 s, and N 1 s spectra. In the C 1 s XPS spectra (Figure 4a), five peaks at 284.8, 286.1, 287.3, 288.6, and 290.0 eV can be relative to C-C/C=C, C-N, C-O, O=C-O, and π-π*, respectively. This shows that this is a typical carbon material structure. In the N 1 s XPS spectra (Figure 4b), four peaks at 398.6, 400.3, 401.5, and 403.1 eV were attributed to pyridinic-N, pyrrolic-N, graphitic-N, and oxidized-N, respectively. These nitrogen-containing components mainly come from self-doping of SH and form specific alkaline functional groups under the action of K_2_FeO_4_. It has been reported that pyridinic-N is found in 6-membered ring structures and that pyrrolic-N is found in 5-membered ring structures [42]. Pyridinic-N contributes one electron to the aromatic π-system, and pyrrolic-N contributes two p-electrons to the π-system. Therefore, they have a Lewis basic character and are beneficial for the capture of CO_2_ (Lewis acid) [43]. In the O 1 s XPS spectra (Figure 4c), four peaks at 530.5, 531.9, 533.5, and 535.5 eV were attributed to C=O, C-O-C/O=C-O, C-OH, and H_2_O-O_2_, which exist in the form of carbonyl or ketone groups (C=O), carbonyl oxygen of esters, anhydrides, amides, and oxygen atoms of phenol and alcohol or ether groups (C-O-C/O=C-O), oxygen of carboxylic groups (C-OH), and oxygen in water, respectively. As shown in Table 2, C=O and C-O-C/O=C-O account for 6.74% and 58.95% in 25%-ASHC. According to the literature [44], they have basicity, which is more conducive to the adsorption of the acid gas CO_2_.

#### 2.1.5. XRD Analysis

**Figure 5:**
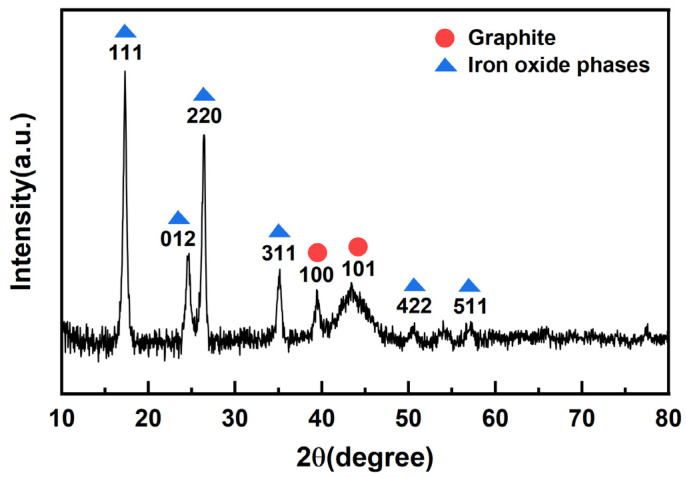
XRD pattern of 25%−ASHC.

The X-ray diffraction pattern of 25%-ASHC is shown in Figure 5. Sharp diffraction peaks can be observed, indicating the presence of large crystallite size for iron species (e.g., α-Fe_2_O_3_ (JCPDS No. 33-0664), γ-Fe_2_O_3_ (JCPDS No. 39-1346), Fe_3_O_4_ (JCPDS No. 19-0629)) [44]. Both Fe_3_O_4_ and γ-Fe_2_O_3_ have the very same cubic spinel crystal structure and similar d-spacing values, thus making it difficult to distinguish from each other by XRD patterns. The characteristic peaks with degrees of 17.26°, 26.39°, 35.07°, 50.62°, and 57.11° correspond to the (111), (220), (311), (422), and (511) plane of Fe_3_O_4_ and/or γ-Fe_2_O_3_, respectively. This is very different from the carbon materials we previously obtained via KOH activation. The peak with the degree of 24.60° corresponds to the (012) plane of α-Fe_2_O_3_, which may conceal the (002) plane diffraction peak of carbon material. The characteristic peaks with degrees of 39.36° and 43.42°correspond to the (100) and (101) plane of graphite, respectively, indicating that the activated carbon materials have a certain tendency to graphitize, which is conducive to improving the stability of carbon materials that is due to the catalytic graphitization of Fe-based components. We can also see the trend of graphitization in further RAM characterization.

#### 2.1.6. RAM Analysis

**Figure 6:**
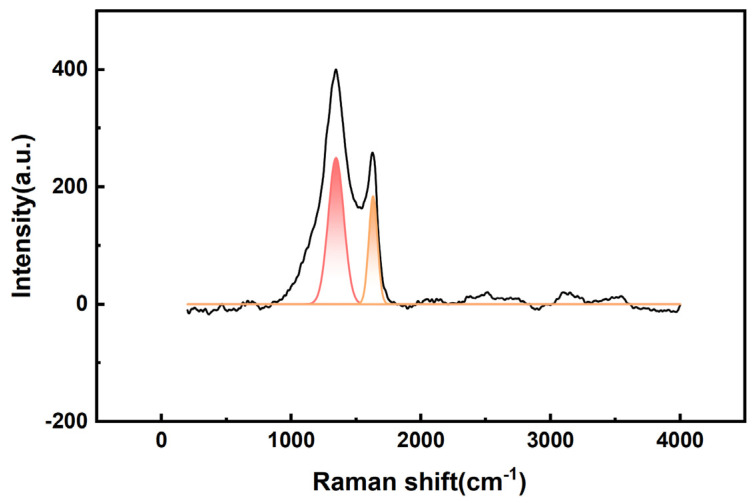
Raman spectra of 25%−ASHC.

The Raman spectra of 25%-ASHC is shown in Figure 6. The D band near 1345 cm^−1^ belongs to the A_1g_ mode, corresponding to defective sites or disordered sp2 hybridized carbon atoms of graphite, which is usually used to reflect the degree of defects or crystallinity of the carbon material. The G band near 1630 cm^−1^ belongs to the E_2g_ mode, which corresponds to the vibrating atoms in the phonon mode plane of the sp2-bonded carbon, reflecting the degree of graphitization of the carbon material. The intensity ratio of the D band to the G band, I_D_/I_G_ = 1.36, indicates that 25%-ASHC has a certain trend of graphitization. However, compared with other activated carbons with higher degrees of graphitization (I_D_/I_G_ < 1), it may also have an amorphous structure of disorganization due to the abundance of oxygen-containing functional groups (e.g., carboxyl groups) on the surface that are destructive to the graphite structure [45,46].

### 2.2. CO_2_ Adsorption Capacity

#### 2.2.1. CO_2_ Uptake Capacity with Different Temperatures

**Figure 7:**
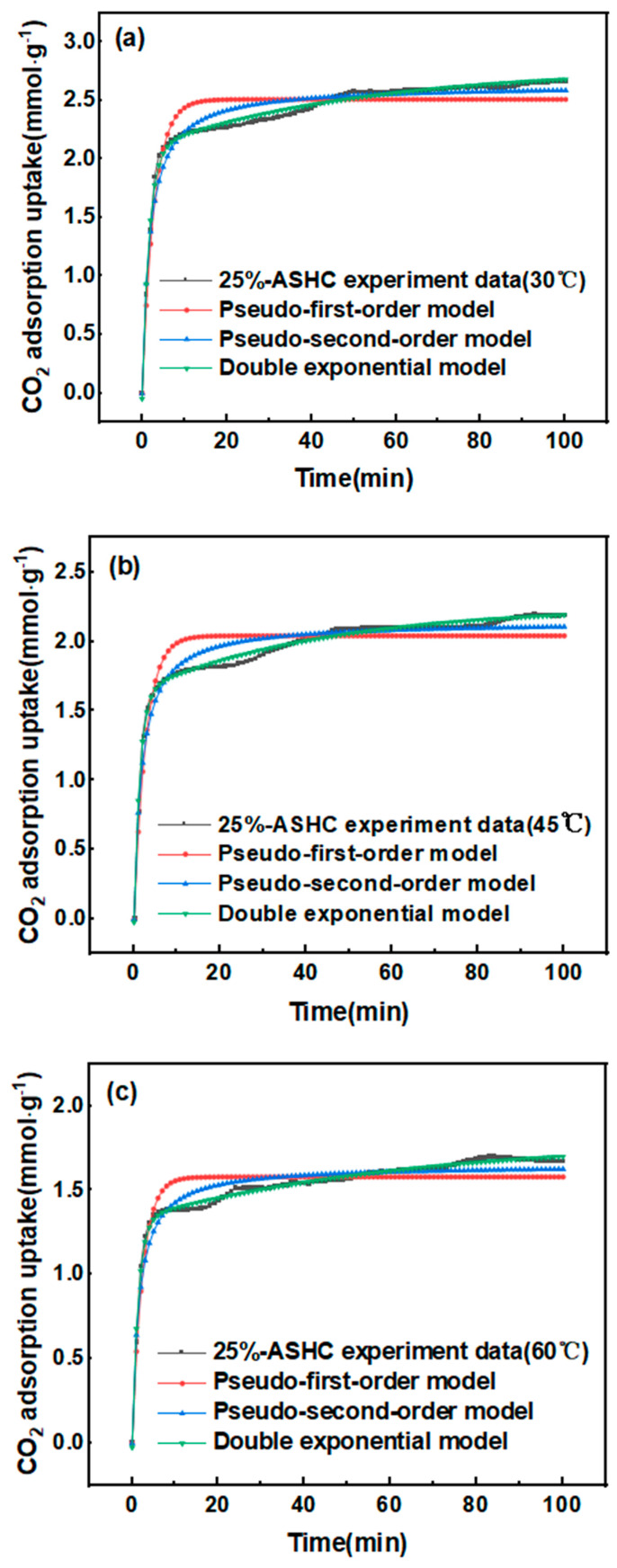
Experimental- and model-predicted values of CO_2_ adsorption of 25%−ASHC at (**a**) 30 °C; (**b**) 45 °C; (**c**) 60 °C.

The CO_2_ adsorption capacities of 0%-SHC and 25%-ASHC at 30, 45, 60 °C, and 1 bar are shown in Table 3, which are derived from the maximum adsorption amount at the end of adsorption in Figure 7. As we can see, 25%-ASHC showed higher CO_2_ adsorption capacities of 2.67 mmol·g^−1^, 2.17 mmol·g^−1^, and 1.70 mmol·g^−1^, which were all higher than 0%-SHC under the same conditions. This result indicated that the CO_2_ adsorption capacity of activated carbon can be significantly enhanced by the addition of activators with maximum CO_2_ adsorption capacities when the incorporation of K_2_FeO_4_ was 25wt%, which was primarily attributed to its highest specific surface area and biggest microporous volume. Though SH and all ASHCs have almost the same functional groups on the surface, further CO_2_ adsorption was limited by their lower porosity and smaller micropore volume as a consequence of too little or too much dosage of K_2_FeO_4_. This also indicates that the activation process greatly enhanced the CO_2_ physical adsorption. Additionally, the adsorption capacity of the samples was found to decrease with increasing temperature, as shown in Table 3. This is because both the surface energy and molecular diffusion of CO_2_ on the sample surface increased, making CO_2_ molecules easier to desorb while the temperature increased.

A comparison of the textural properties and adsorption performance with biomass-activated carbon prepared by other methods is shown in Table 4. It can be seen that the ASHC had a considerable CO_2_ adsorption capacity over other carbon materials. It shows that the synthesis of activated carbon by one-step method using K_2_FeO_4_ as a catalytic oxidative activator and SH as a precursor is an efficient method for the preparation of activated carbon.

#### 2.2.2. Adsorption Kinetics with Different Temperatures

The CO_2_ adsorption curves of 25%-ASHC at 30, 45, and 60 °C are shown in Figure 7. The CO_2_ adsorption reached 50% of the equilibrium capacity within 1 min and 80% of the adsorption equilibrium within 6 min after the start of the CO_2_ gas flow, indicating a rapid rate of CO_2_ adsorption. Three kinetic models, namely pseudo-first-order model, pseudo-second-order model, and double exponential model, were used to further investigate the adsorption kinetics. The accuracy of these kinetic models in predicting the adsorption capacity was also assessed based on R^2^ (linear regression coefficient). As shown in Figure 7, the double exponential model had the best fitness for CO_2_ adsorption capacity of 25%-ASHC at 30 °C, 45 °C, and 60 °C due to its highest value of R^2^ (>99%), while other methods had values less than 95%; the relevant kinetic parameters are listed in Table 5. Thus, the double exponential kinetic model provided the best description over the entire adsorption process. It suggests that physical adsorption and chemical adsorption occur simultaneously [55]. This is also consistent with the previous BET, FTIR, and XPS test results; the pore structure and the abundant functional groups on its surface both contribute to the high carbon dioxide adsorption performance. According to the fitting results shown in Table 5, A_1_ was bigger than A_2_, which means that physical adsorption is stronger than chemical adsorption. At the same time, A_1_ decreased signifcantly and A_2_ only decreased by a small amount when the temperature increased. This is because at the first adsorption site, physical adsorption decreases significantly with an increase in temperature, while the physical adsorption is based on the Langmuir adsorption model. The lower the temperature, the greater the adsorption capacity, which is due to the Langmuir adsorption constant increasing with a decrease in temperature. Meanwhile, at the second adsorption site, chemical adsorption is not very dependent on temperature; its strength does not change much with an increase in temperature.

#### 2.2.3. Adsorbent Regeneration

**Figure 8:**
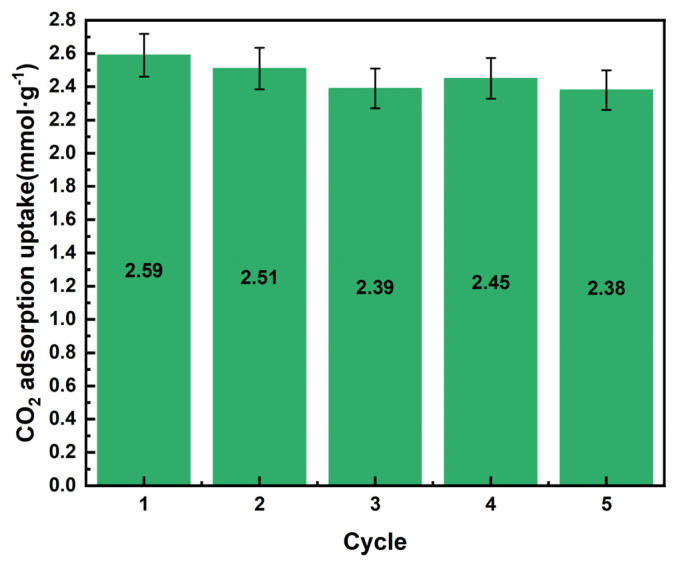
CO_2_ adsorption−desorption cycle curves of 25%−ASHC.

Five consecutive adsorption-resolution cycle curves of 25%-ASHC are shown in Figure 8. A slight increase in the maximum adsorption capacity was noticed in the fourth cycle, which can be explained by the redistribution of the products and regeneration during the cycle, which allowed the unreacted chemical sites to react in contact with the CO_2_ [56]. This phenomenon is stochastic in nature. In general, the adsorption capacity remained above 92% after five cycles, demonstrating that the adsorbent not only has good adsorption performance, but also has good regeneration performance and strong cyclic stability. Consequently, the prepared ASHCs have the potential to be reused several times in the CO_2_ capture process. Additionally, the regeneration could be achieved by temperature desorption at 130 °C for 10 min, making the process feasible and economical.

## 3. Materials and Methods

### 3.1. Materials

SH was collected from Wenzhou coastal area in Zhejiang Province, PR China. After series pretreatment procedures, including washing, drying, crushing and sieving, SH particles with a mean diameter of 40 mesh were provided for porous carbon preparation.

Potassium ferrate (K_2_FeO_4_, CAS 39469-86-8, AR, 99%) was purchased from Shanghai Macklin Biochemical Co. Ltd. (Shanghai, China). Hydrochloric acid (HCl, CAS 7641-01-0, 36.0 wt%) was purchased from Sinopharm Ltd. (Hangzhou, China). Nitrogen (N_2_ CAS 7727-37-9, 99.9%) and CO_2_ (CAS 124-38-999, 99%) gases were purchased from Hangzhou Special Gas Co., Ltd. (Hangzhou, China).

### 3.2. Preparation of Samples

Potassium ferrate and dried SH were mixed by continuously stirring in 10 mL water with different mass ratios and were impregnated for 1 h. The resulting mixture was dried at 105 °C for 12 h and then transferred to a crucible, followed by pyrolysis in a horizontal stainless tube furnace under 750 °C with a heating rate of 10 °C·min^−1^ for 1h. After cooling down, the product was carefully washed with water and then filtered until pH = 8. The generated carbon was again washed with dilute HCl (1 M, 20 mL, stirring with rotor for 3 h) to eliminate K species and other metal irons, and then it was ultrasonically washed with distilled water for 30 min before thorough washing with vacuum filtered until achieving a neutral pH level. Finally, the obtained samples were dried at 105 °C overnight. The resultant samples were denoted as m-ASHC, where m is the mass ratio of K_2_FeO_4_ to SH (they are 5%, 15%, 25%, 50%, and 100%, respectively); samples without added K_2_FeO_4_ but with pyrolysis under 750 °C were defined as 0%-SHC.

### 3.3. Characterization of Samples

The specific surface area and pore volume were determined using a fully automatic surface area analyzer (3H-2000PS1, BSD, Beijing, China), in which the specific surface area was evaluated with the BET method and determined in the partial pressure (P/P_0_) range 0.04–0.32, and total pore volume (V_total_) was determined from the amount of nitrogen adsorbed at a relative pressure of 0.99. The micropore volumes (V_micro_) were calculated by t-plot analysis, and the pore size distributions were obtained according to the Brunauer–Emmet–Teller theory by using the NonLocal Density Functional Theory (NLDFT) method for accurate micropore filling mechanisms.

The morphologies and microstructures of the samples were characterized using the following facilities: a scanning electron microscope (SEM; Vega3, Tescan, Dortmund, Germany) operating at 5.0 kV and 15.0 kV; a transmission electron microscope (TEM; Tecnai G2 F30 S-Twin, Philips-FEI, Hillsboro, OR, USA) with an acceleration voltage of 300 kV; a Nicolet 6700 FTIR spectrometer averaging 24 scans in the 4000–400 cm^−1^ spectral range at 4 cm^−1^ resolution and a KBr pellet used as a reference sample; an X-ray photoelectron spectroscope (XPS; Thermo Scientific K-Alpha, Waltham, MA, USA) whose binding energies were standardized by the C 1 s peak at 284.8 eV; an X-ray diffraction spectrometer (X’Pert Pro, PANalytical, Almelo, The Netherlands) with Cu Kα radiation (λ = 1.5404 Å) over a 10–80° 2θ range and a position-sensitive detector with 0.05° step size at a scan rate of 1 °·min^−1^; a laser scanning confocal micro-Raman spectrometer (LabRAM HR, HORIBA, Palaiseau, France) with a laser excitation wavelength of 488 nm, scanning in an extended range of 0–4000 cm^−1^.

### 3.4. CO_2_ Adsorption Measurements

The CO_2_ adsorption performance of the carbon samples was measured using a thermogravimetric analyzer (TGA 209 F3 Tarsus, NETZSCH, Selb, Germany). Initially, about 10 mg of each sample was placed in an alumina crucible loaded in a TGA furnace. Prior to each adsorption experiment, the carbon sample was heated up to 130 °C (10 °C·min^−1^) and kept for 30 min to remove moisture under N_2_ flow (30 mL·min^−1^). Then, the carbon sample was cooled to a desired adsorption temperature, i.e., 30 °C, 45 °C, and 60 °C, then the CO_2_ adsorption studies were performed for 100 min with CO_2_ flow rate of 50 mL·min^−1^. Moreover, adsorbent regeneration was carried out by heating the sample to 130 °C for 30 min at 10 °C·min^−1^ under N_2_ flow (30 mL·min^−1^). To check the adsorbent stability, the adsorption–desorption procedure was repeated five times for 60 min/time at 30 °C with other conditions remaining the same.

### 3.5. CO_2_ Adsorption Kinetic Analysis

Three typical kinetic models, namely pseudo-first-order model, pseudo-second-order model, and double exponential model were studied in this research. The regression coefficient (R^2^) was verified according to the fitting degree of each theoretical model and the actual data. The highest regression coefficient indicates the most appropriate theoretical model, which can demonstrate the adsorption kinetic mechanism. The three models describe different adsorption mechanisms, respectively.

The pseudo-first order model is based on the assumption that the rate of adsorption is proportional to the primary of the number of possible adsorption sites, primarily used to describe the physical adsorption process. The equation can be expressed as follows:

(12) $$\frac{\partial q_{t}}{\partial t}=k_{1}\left(q_{e}-q_{t}\right)$$

where *q_e_* and *q_t_* denote the amount adsorbed at equilibrium and at a specific time *t* (mmol·g^−1^), respectively; *t* is the adsorption time (min), and *k*_1_ is the pseudo-primary adsorption rate constant (min^−1^). By applying the boundary conditions of *q_t_* = 0 at *t* = 0 and *q_t_* = *q_e_* at *t* = ∞, the integral form of the equation can be expressed as follows:

(13) $$q_{t}=q_{e}\left(1-e^{-k_{1}t}\right)$$

The pseudo-second-order model is based on the assumption that the rate of adsorption is proportional to the square of the number of possible adsorption sites, mainly used to describe the chemisorption processes. The equation can be expressed as follows:

(14) $$\frac{\partial q_{t}}{\partial t}={k_{2}\left(q_{e}-q_{t}\right)}^{2}$$

where *q_e_* and *q_t_* denote the amount adsorbed at equilibrium and at a specific time *t* (mmol·g^−1^), respectively; *t* is the adsorption time (min), and *k*_2_ is the pseudo-secondary adsorption rate constant (g/(mmol·min)). By applying the boundary conditions of *q_t_* = 0 at *t* = 0 and *q_t_* = *q_e_* at *t* = ∞, the integral form of the equation can be expressed as follows:

(15) $$q_{t}=\frac{1}{\frac{1}{k_{2}{q_{e}}^{2}t}+\frac{1}{q_{e}}}$$

The double exponential model is primarily used to describe adsorption processes where both physical and chemical adsorption coexist. The equation can be expressed as follows:

(16) $$q_{t}=q_{e}-A_{1}e^{-k_{3}t}-A_{2}e^{-k_{4}t}$$

where *q_e_* and *q_t_* denote the amount adsorbed at equilibrium and at a specific time *t* (mmol·g^−1^), respectively; *t* is the adsorption time (min), and *k*_3_, *k*_4_ are the kinetic rate constants for physical adsorption and chemical adsorption of the CO_2_ (min^−1^), respectively; A_1_, A_2_ are similar to the amount of CO_2_ adsorbed at equilibrium after physical adsorption and chemical adsorption, respectively. This model is considered in this study because of its feasibility in explaining the adsorption kinetics for an adsorbent that possesses two different adsorption sites. This model also offers an advantage of explaining kinetic mechanisms which involve two steps, namely a rapid phase controlled by physical adsorption and a slow phase controlled by chemical adsorption. The major merit of this model is that it takes into account surface heterogeneity in a similar way to the Langmuir dual site.

## 4. Conclusions

In this study, porous carbon with high adsorption properties was prepared by one-step pyrolysis using SH as precursor and K_2_FeO_4_ as activator under 750 °C. The porous carbon could be obtained with a highest specific surface area of 1245 m^2^·g^−1^ and a highest CO_2_ adsorption capacity of 2.67 mmol·g^−1^ at 30 °C and 1 bar, with excellent cycling stability and easy regeneration when the mass ratio of K_2_FeO_4_ and SH was 25 wt%. Compared to our previous carbon dioxide adsorbent prepared by KOH activation, its performance in adsorption and regeneration were both enhanced with a faster rate, although the specific surface area was diminished. Meanwhile, this work replaced abundant KOH with less K_2_FeO_4_, making the reaction more efficient and economical. The contribution of the “trinity” activation of K_2_FeO_4_ can be divided into three aspects: (1) activator: synergizing with the metal ions contained in SH, such as K, Ca, Na, etc., to promote the formation of pore structure; (2) oxidant: using the strong oxidative property of Fe(VI), the porous carbon surface forms oxygen-containing functional groups, which improves the adsorption performance of CO_2_; (3) catalyst: playing the role of catalytic acceleration so that the reaction can be carried out rapidly at lower temperatures to reduce the preparation time and energy consumption, which is conducive to the large-scale production of products. In general, using K_2_FeO_4_ oxidative pyrolysis SH proved to be an efficient way to prepare adsorbent for CO_2_ capture. This simple and cost-effective carbon synthesis route is beneficial to the large-scale preparation of CO_2_ adsorbent in dealing with the problem of the greenhouse effect. It can also turn waste golden tide into treasure in order to promote the development of the ecological restoration industry for macroalgae.

## Figures and Tables

**Table 1:** Textural properties of samples.

Adsorbents	Surface Area	Pore Volume			Average Pore Diameter (nm)	Microporous Average Pore Diameter (nm)
	S_BET_ (m^2^·g^−1^)	V_total_ (cm^3^·g^−1^)	V_micro_ (cm^3^·g^−1^)	V_micro_ /V_tolal_		
0%-SHC	30.3827	0.0446	0.0133	0.2982	5.8718	1.2198
5%-ASHC	297.1456	0.2046	0.1049	0.5127	2.7542	0.6864
15%-ASHC	417.9733	0.3352	0.1539	0.4591	3.2079	0.6570
25%-ASHC	1245.0812	0.8827	0.5682	0.6437	2.8358	0.7266
50%-ASHC	730.5373	0.6926	0.3284	0.4742	3.7923	0.7994
100%-ASHC	586.6224	0.4702	0.2379	0.5060	3.2062	0.7480

**Table 2:** Peak area ratios of O 1 s.

Sample	C=O	C-O-C/O=C-O	C-OH	H_2_O-O_2_
25%-ASHC	6.74	58.98	28.51	5.77

**Table 3:** CO_2_ adsorption capacity of samples at different adsorption temperatures.

Samples	CO_2_ Adsorption Capacity (mmol·g^−1^)		
	30 °C	45 °C	60 °C
0%-SHC	0.94	0.78	0.63
25%-ASHC	2.67	2.17	1.70

**Table 4:** Comparison of textural properties and CO_2_ adsorption performance.

Feedstock	Activation	S_BET_ (m^2^·g^−1^)	V_total_ (cm^3^·g^−1^)	V_micro_ (cm^3^·g^−1^)	CO_2_ Uptakes (mmol·g^−1^)		Ref.
					25 °C/1 bar	30 °C/1 bar	
Wooden chopstick	KOH	N/A	N/A	N/A	2.63	N/A	[47]
Dried rice husk	KOH/PEI	1190	0.777	0.422	1.90	N/A	[48]
Arundo donax	KOH/ZnCl_2_	982	0.62	N/A	2.20	N/A	[49]
Palm date seeds	H_3_PO_4_	1439	0.60	N/A	4.40	N/A	[50]
Walnut shell	Mg(NO_3_)_2_	292	0.157	0.118	1.86	N/A	[51]
Coconut	H_3_PO_4_	1322	0.61	0.49	N/A	3.7	[52]
Rice husk	ZnCl_2_/HCl	927	0.56	N/A	N/A	1.3	[53]
Nypha fruticans	Mg(NO_3_)_2_/Cu(NO_3_)_2_	727.7	0.50	0.26	N/A	1.91	[54]
*Sargassum horneri*	K_2_FeO_4_	1245	0.8827	0.5682	N/A	2.67	This work

**Table 5:** The kinetic parameters of CO_2_ adsorption of 25%-ASHC.

Kinetic Models	Parameters	Adsorption Temperatures		
		30 °C	45 °C	60 °C
Pseudo-first-order	*q*_m,exp_ (mmol·g^−1^)	2.67	2.17	1.70
	*q*_e,cal_ (mmol·g^−1^)	2.51	2.04	1.58
	*k*_1_ (min^−1^)	0.3549	0.3668	0.4229
	*R* ^2^	0.8499	0.8029	0.8190
	*E%*	2.87	2.66	1.90
Pseudo-second-order	*q*_e,cal_ (mmol·g^−1^)	2.63	2.14	1.65
	*k*_2_ (g/(mg·min))	0.2092	0.2577	0.3860
	*R* ^2^	0.9453	0.9283	0.9287
	*E*%	2.63	5.68	1.57
	*q*_e,cal_ (mmol·g^−1^)	2.78	2.25	1.80
Double Exponential	A_1_	2.1343	1.6579	1.3535
	*k*_3_	0.6026	0.7312	0.7211
	A_2_	0.6903	0.6208	0.4737
	*k*_4_	0.0197	0.0227	0.0157
	*R* ^2^	0.9913	0.9905	0.9920
	*E*%	1.60	3.58	4.47

## Data Availability

The data used to support the findings of this study are included within the article.

## Supplementary Materials

The following supporting information can be downloaded at: https://www.mdpi.com/article/10.3390/molecules29061345/s1, Figure S1: SEM image of (a) 15%-ASHC; (b) 50%-ASHC; Figure S2. XPS spectrum of 25%-ASHC.
