# Supplementary figures and images for: Turn Waste Golden Tide into Treasure: Bio-Adsorbent Synthesis for CO2 Capture with K2FeO4 as Catalytic Oxidative Activator
Source: Molecules. 2024 Mar 18;29(6):1345. doi: 10.3390/molecules29061345 (PMC10975329; doi:10.3390/molecules29061345)

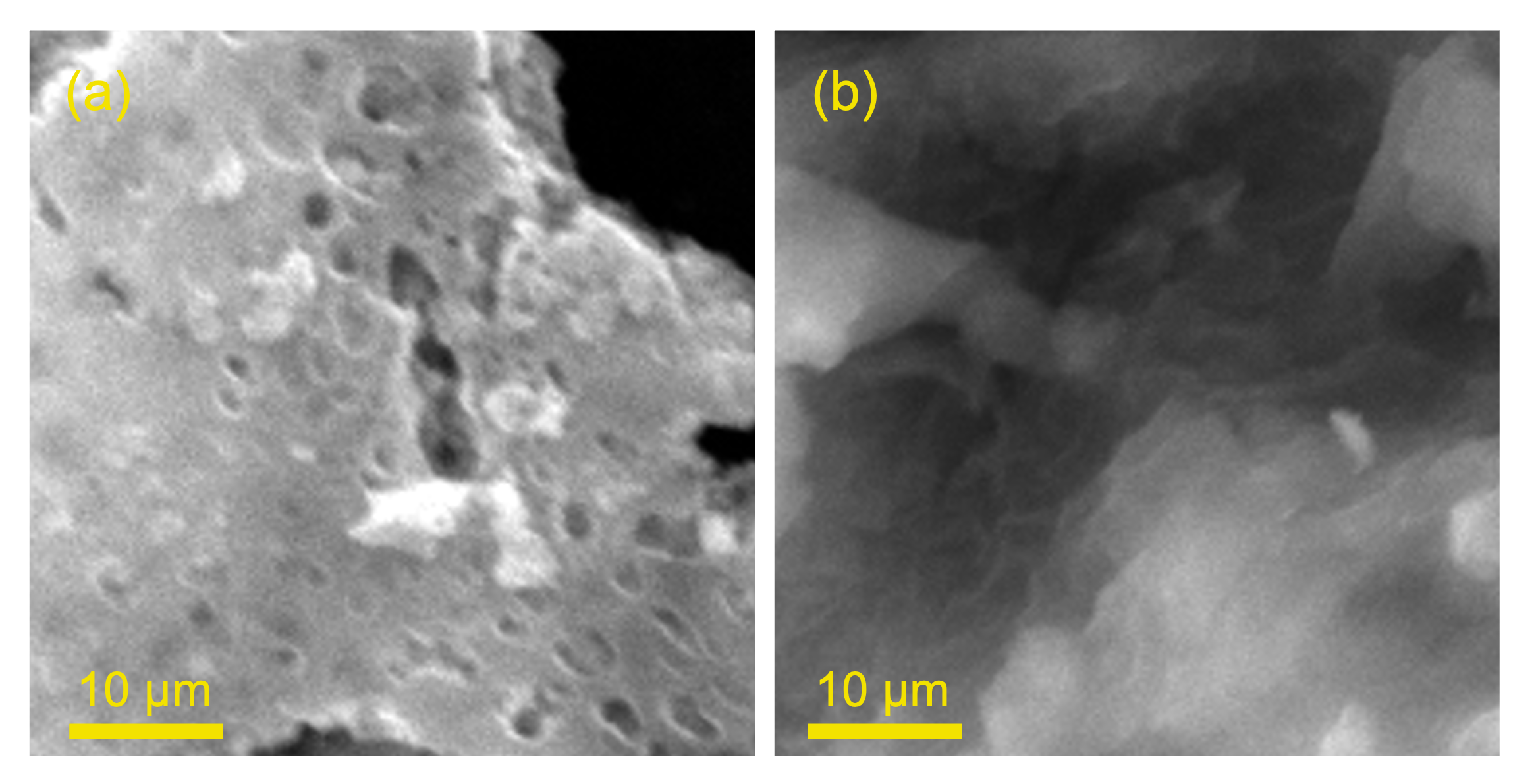

Supplement: Supplementary file 1 [file molecules-29-01345-s001.zip › S1 2.20/S1.SEM image of (a)15%-ASHC;(b)50%-ASHC.png]

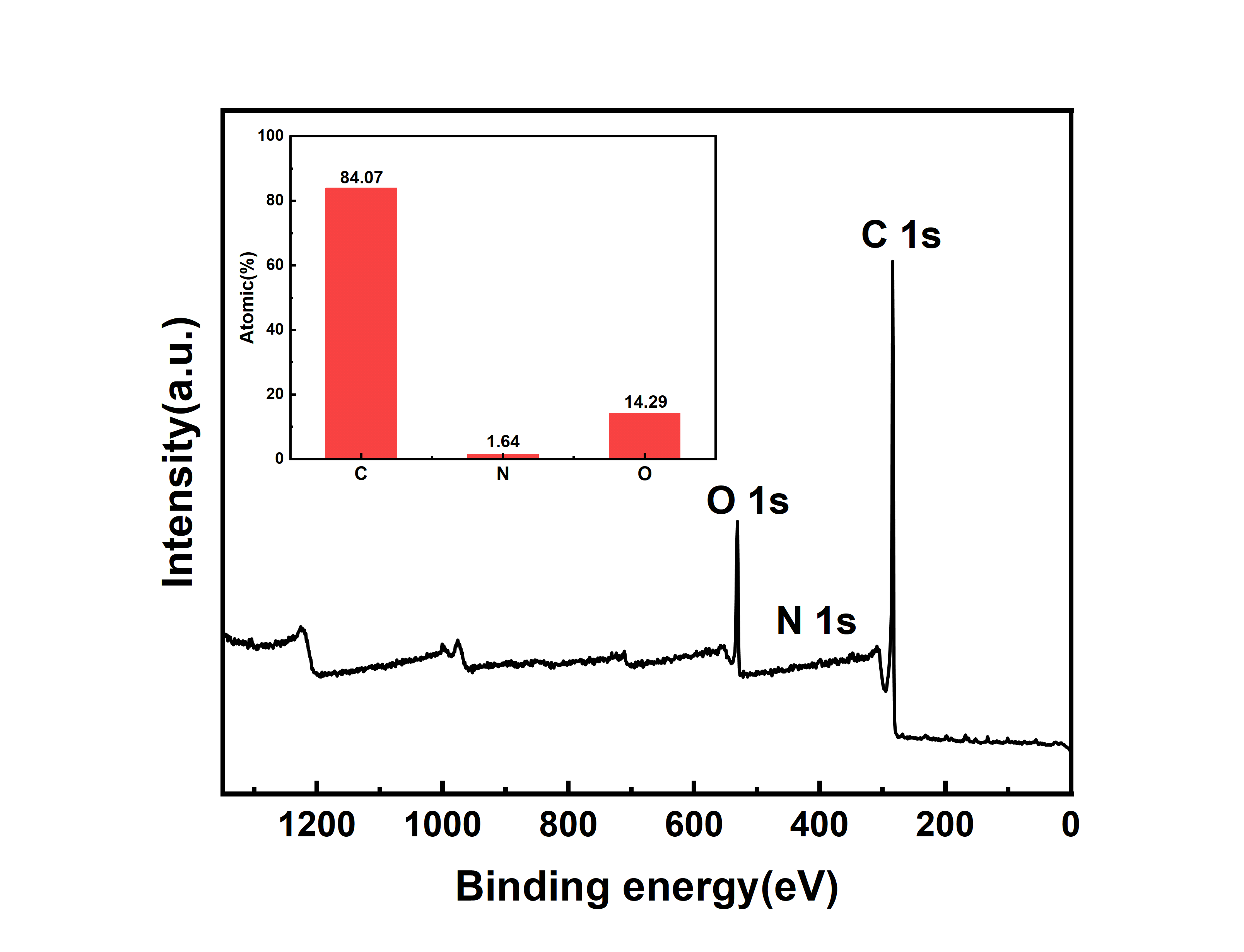

Supplement: Supplementary file 1 [file molecules-29-01345-s001.zip › S1 2.20/S2. XPS full spectrum of 25%-ASHC.png]
